# Assessment of Toxicological Perturbations and Variants of Pancreatic Islet Development in the Zebrafish Model

**DOI:** 10.3390/toxics4030020

**Published:** 2016-09-02

**Authors:** Karilyn E. Sant, Haydee M. Jacobs, Jiali Xu, Katrina A. Borofski, Larry G. Moss, Jennifer B. Moss, Alicia R. Timme-Laragy

**Affiliations:** 1 Department of Environmental Health Sciences, School of Public Health and Health Sciences, University of Massachusetts Amherst, Amherst, MA 01003, USA; ksant@umass.edu (K.E.S.); hmjacobs@umass.edu (H.M.J.); jialix@umass.edu (J.X.); kborofski@umass.edu (K.A.B.); 2 Duke Molecular Physiology Institute, Endocrine Division, Duke University Medical Center, Durham, NC 27701, USA; larry.moss@dm.duke.edu (L.G.M.); jennifer.b.moss@duke.edu (J.B.M.)

**Keywords:** endocrine pancreas, pancreatic toxicology, beta cell, zebrafish, exocrine, endocrine disrupting chemicals, morphology

## Abstract

The pancreatic islets, largely comprised of insulin-producing beta cells, play a critical role in endocrine signaling and glucose homeostasis. Because they have low levels of antioxidant defenses and a high perfusion rate, the endocrine islets may be a highly susceptible target tissue of chemical exposures. However, this endpoint, as well as the integrity of the surrounding exocrine pancreas, is often overlooked in studies of developmental toxicology. Disruption of development by toxicants can alter cell fate and migration, resulting in structural alterations that are difficult to detect in mammalian embryo systems, but that are easily observed in the zebrafish embryo model (*Danio rerio*). Using endogenously expressed fluorescent protein markers for developing zebrafish beta cells and exocrine pancreas tissue, we documented differences in islet area and incidence rates of islet morphological variants in zebrafish embryos between 48 and 96 h post fertilization (hpf), raised under control conditions commonly used in embryotoxicity assays. We identified critical windows for chemical exposures during which increased incidences of endocrine pancreas abnormalities were observed following exposure to cyclopamine (2–12 hpf), Mono-2-ethylhexyl phthalate (MEHP) (3–48 hpf), and Perfluorooctanesulfonic acid (PFOS) (3–48 hpf). Both islet area and length of the exocrine pancreas were sensitive to oxidative stress from exposure to the oxidant tert-butyl hydroperoxide during a highly proliferative critical window (72 hpf). Finally, pancreatic dysmorphogenesis following developmental exposures is discussed with respect to human disease.

## 1. Introduction

Developmental exposures to environmental toxicants can be highly disruptive to embryonic development, producing outcomes ranging from embryonic lethality and congenital malformations, to subtle physiological or morphological alterations that may predispose individuals to diseases that emerge later in life [1,2,3,4]. Classically, developmental studies have focused on teratogenic outcomes, with many of these effects arising from public crises such as those caused by diethylstilbestrol and thalidomide. However, it is likely that the vast majorities of in utero exposures are less severe and produced by lower-dose exposures of environmental contaminants. While these outcomes could manifest pathologically during childhood, these exposures may instead increase fragility of tissues and signaling pathways, and increase susceptibility to future stresses or exposures acquired throughout the lifecourse. This is a fundamental component of the developmental origins of health and disease paradigm, which postulates that later-life effects and chronic diseases such as diabetes could be caused by early-life conditions, including chemical exposures [5].

Diabetes is a human health problem of increasing concern, with high economic and societal cost [6]. Rates of both Type 1 and Type 2 diabetes have increased more than can be predicted by genetics alone, suggesting they may be associated with environmental factors, such as chemical exposures [1,7]. While Type 1 and Type 2 are distinct diseases with differing causal pathologies, both manifest common aspects of the diabetic phenotype that are related to pancreatic beta cells, particularly elevated blood glucose, largely attributed to insufficient signaling of insulin, and compromised beta cell integrity. Several environmental toxicants have been associated with the development of diabetes in epidemiological studies, or found to disrupt pancreatic beta cells in animal models. This diverse group of toxicants includes polychlorinated biphenyls/dioxins, phthalates, perfluorinated compounds, and pharmaceuticals [8,9,10,11,12,13,14]. However, a direct causal link between embryonic exposure and the development of diabetes or metabolic diseases later in life has not yet been firmly established.

Zebrafish pancreas and beta cell development have been extensively characterized and human relevance firmly established [15,16,17,18,19,20]. In vivo observation of endocrine pancreas development has been accomplished by many groups, and high throughput drug screens using developing zebrafish as targets for tissue specific changes during morphogenesis have identified pharmaceuticals that may be effective in alleviating human disease [21]. Many toxicology studies have investigated the association between xenobiotics and aggressive adult pancreas diseases, such as pancreatitis and pancreatic cancer, but studies investigating prenatal toxicant exposures to the developing pancreas and their consequences are especially challenging due to the invasiveness of probing in utero conditions and the duration of time until disease prognosis. Therefore, the zebrafish, which have transparent embryos, are an ideal model for these studies.

There are important physiological reasons why the endocrine islets are a target of toxicants during development. First, pancreatic beta cells have low levels of antioxidant defenses [22,23] and are exquisitely sensitive to chemical-induced oxidative stress [24]. This has important implications during vertebrate islet development since oxidative stress can divert progenitor cells from a proliferative state to one of premature differentiation [10], affecting beta cells as well as other islet cells including alpha cells. Second, beta cells are arranged in islets that are densely vascularized to facilitate optimal functioning of the islets. In order to regulate insulin synthesis and release, beta cells require a highly perfused environment to sense blood glucose levels and release insulin to the blood stream to maintain glucose homeostasis in the body. The interaction of beta cells with the islet vasculature during development, and in diseases such as Type 2 diabetes that have been described as a microvascular disease, is crucial to maintaining islet function and health [25]. The highly vascular nature of the islets and perfusion of islet capillaries would simultaneously allow for proficient drug/toxicant delivery.

Though the endocrine pancreas is a key regulator of energy homeostasis, the majority of the pancreas is comprised of exocrine tissue. Islets are embedded into the exocrine tissue, which is a vast network of ducts responsible for producing and secreting bicarbonate and digestive peptides such as proteases and lipases. Exocrine pancreas insufficiency is defined as malnutrition due to deficiencies in the secretion of these digestive products, and can occur following pathologies such as pancreatitis or even congenitally shortened pancreas (dorsal pancreas agenesis) [26]. Infants born with severe dorsal pancreatic agenesis are prone to neonatal diabetes and severe exocrine insufficiency [27]. While a congenitally shortened pancreas is likely to directly influence exocrine function, it is also possible that this decrease in total pancreas length may have endocrine consequences. Epidemiological evidence suggests that diabetics are more likely to have shorter pancreas lengths [28]. Though islets exist throughout the body of the pancreas, the majority of islets are concentrated posteriorly in the body and tail regions of the pancreas [29,30,31]. Thus, shortened pancreas length could potentially impact the number of islets due to decreased habitable area.

Toxicology studies must rely on well-characterized controls due to the variability of responses observed in living models. Many fish embryotoxicity assays use standard control conditions characterized by the use of the solvent dimethyl sulfoxide (DMSO) to solubilize and deliver hydrophobic toxicants to the target organism. DMSO, when used at low, non-toxic concentrations, has negligible biological effects. However, the prevalence of islet variants observed in DMSO control embryos in our previous study [14] required further investigation. Population or inter-individual variability is an important consideration for toxicological studies, as a range of responses is often observed. This variability should be used to inform toxicological studies about the appropriateness of statistical models and sample sizes, and has yet to be characterized for the embryonic zebrafish islet.

In this exploratory study, we examined islet development during different developmental windows, following toxicological challenge with several chemical challenges. Cyclopamine is an environmental toxin found in the Western corn lily. This steroidal alkaloid has been shown to cause cyclopia in livestock [32]. Cyclopamine is a general inhibitor of Hedgehog action that interferes with Smoothened, a plasma membrane protein that interacts with the Patched receptor [15]. Mono-2-ethylhexyl phthalate (MEHP) is the primary metabolite of di-2-ethylhexyl phthalate (DEHP), a ubiquitous plasticizer in polyvinyl chloride piping, food packaging, medical devices, and toys [33]. MEHP has been demonstrated to cross the placenta, and has been detected in breast milk and cord blood [34]. MEHP can also activate peroxisome proliferator-activated receptor (PPAR) gamma, and may alter processes such as embryonic nutrient uptake and utilization which influence metabolic programming [35,36,37]. Perfluorooctanesulfonic acid (PFOS) is a surfactant used in Teflon^TM^ and Scotchguard^TM^ until 2000, and has been universally detected in human tissues, including cord blood samples [38], amniotic fluid [39,40,41], and adult pancreas [42]. *Tert* butylhydroperoxide (tBOOH) is an organic peroxide that causes oxidative damage. Several of these chemicals, including PFOS and MEHP, have been associated with Type 2 diabetes in human epidemiology studies, as well as altered beta cell structure and function and glucose homeostasis across multiple models [9,12,33,43,44].

Our work builds on experimental paradigms established by previous studies where we and others have used the developing pancreas marked with fluorescent beta cells as a screening tool to identify nutritional or pharmaceutical agents that could be repurposed to increase the number of beta cells for therapeutic discoveries relevant to diabetes. Results of such studies have shown significant effects on the developing endocrine pancreas including precocious formation of secondary islets [10,21,45,46] and changes in beta cell proliferation and regeneration [21,47,48,49,50]. Herein, we establish a baseline of normal islet morphological variants following treatment with the most commonly utilized vehicle control for zebrafish embryotoxicity studies, DMSO, demonstrate the importance of critical windows of exposure during pancreas development, and examine incidence of islet abnormalities that we have observed with exposure to several environmental contaminants.

## 2. Materials and Methods

### 2.1. Fish

This study used transgenic zebrafish, *Tg(ins:GFP)* [15], that expresses green fluorescent protein (GFP) only in the insulin-producing beta cells. Other constructs have been found to be ‘leaky’ and express GFP or Cre recombinase in the hypothalamus, complicating developmental assessments and metabolic measurements [51]. To study effects on the exocrine pancreas, we used *Tg(ptf1a:GFP),* a transgenic fish line that expresses GFP in the exocrine pancreas tissues, the retina, and parts of the brain [52,53]. Both fish lines were obtained as heterozygous populations from Dr. Phillip Di Iorio at the University of Massachusetts Medical School zebrafish facility (Worcester, MA, USA) and bred to homozygosity at the University of Massachusetts Amherst with strict ethical consideration and under the approval of approved institutional protocols (Animal Welfare Assurance Number A3551-01). Adult fish were maintained at 28.5 °C on a 14 h light: 10 h dark cycle in an Aquaneering zebrafish system. Embryos were collected from large breeding tanks, containing approximately 15 males and 15 females, and placed into 0.3 × Danieau’s water (17 mM NaCl, 2 mM KCl, 0.12 mM MgSO_4_, 1.8 mM Ca(NO_3_)_2_, 1.5 mM HEPES, pH 7.6), or “egg water”, for the duration of the experiment.

### 2.2. Chemicals

Cyclopamine was provided for these studies as a generous gift from W. Gaffield. Cyclopamine was prepared in 95% ethanol as a 10 mM stock solution. Monoethylhexyl phthalate (MEHP) was purchased from AccuStandard (New Haven, CT, USA). Heptadecafluorooctanesulfonic acid solution (perfluorooctanesulfonic acid; PFOS) was purchased from Sigma-Aldrich (St. Louis, MO, USA). MEHP and PFOS were prepared as stock solutions in dimethyl sulfoxide (DMSO, Fisher Scientific, Pittsburgh, PA, USA). Solutions were vortexed prior to use and stored at −20 °C in amber glass vials. *Tert* butylhydroperoxide (tBOOH) was purchased from Alfa Aesar, a subsidiary of Fisher Scientific, and stored at 4 °C.

### 2.3. Exposures

To determine whether exposure to the common solvent control DMSO resulted in altered islet development, we exposed replicate groups of five *Tg(ins:GFP)* embryos to 0.01% DMSO or 0.3 × Danieau’s water. We assessed islets in eleutheroembryos (zebrafish that are post-hatch but not yet independent-feeding) at 96 hpf, which is the standard end point in the fish embryo toxicity test.

To characterize potential islet variants with DMSO, we exposed groups of 15 embryos to 0.01% DMSO in 0.3 × Danieau’s water, in glass petri dishes beginning at 3 hpf with daily renewal. Embryos were imaged for brightfield and fluorescent microscopy at 48, 72, 96, and 168 hpf.

To investigate critical windows of islet development, we used the inhibitor of hedgehog signaling, cyclopamine. Homozygous *Tg(ins:GFP)* embryos on an AB background strain [15] were grown at 28 °C on agarose treated dishes in egg water with or without cyclopamine and evaluated with a fluorescence microscope using a GFP2 filter. Chorions were removed using pronase [54]. Cyclopamine was diluted to 50 µM in egg water, and was added to triplicate 10 cm agarose-coated dishes containing 50 homozygous embryos beginning at either 2, 4, 6, 8, or 12 hpf; exposures beginning at 18, 24, and 48 hpf were performed in duplicate. At 72 h post fertilization (hpf), when beta cells have normally coalesced into a single cluster, we recorded the number of *Tg(ins:GFP)* transgenic embryos in each dish which had GFP + fluorescent cells arranged in normal beta cell clusters, ectopic beta cells that resembled an anterior duplication of a single beta cells, stunted islets comprised of single cells, or surviving embryos lacking GFP fluorescence suggesting an absence of beta cells.

To assess a sub-chronic developmental exposure, we used the PPAR-activators and environmental contaminants MEHP and PFOS. Homozygous *Tg(ins:GFP)* embryos were confirmed for successful fertilization before chemical treatment began at 3 hpf. Both MEHP and PFOS were dissolved into DMSO at concentrations so that the total concentration of dosing solution in egg water was 0.01% *v*/*v*, in glass dishes. Embryos were exposed to 0.7 µM MEHP, with exposures renewed every 24 h. Embryos for the PFOS study were exposed to 32 µM PFOS daily, with exposures renewed every 24 h. Embryos were imaged for brightfield and GFP fluorescence at 48 hpf. Each exposure group contained 15–20 embryos, and each experiment was repeated at least three times.

To determine whether pancreas development is sensitive to oxidative stress exposures, homozygous groups of five *Tg(ins:GFP)* and *Tg(ptf1a:GFP)* were exposed to a nontoxic concentration of 77.5 µM tBOOH or water in 0.3 × Danieau’s water for 10 minutes beginning at 72 hpf in a glass petri dish. After the exposure, the eleutheroembryos were transferred to a clean dish and maintained until imaging at either 80 hpf (exocrine pancreas) or 96 hpf (islet). This experiment was repeated at least four times.

### 2.4. Microscopy

Zebrafish embryos and eleutheroembryos and larvae were manually or chemically dechorionated, anesthetized in egg water containing MS-222, and staged in methylcellulose or imaged on agarose dishes. Microscopy for MEHP and PFOS experiments was performed on an EVOS FL Auto imaging system, calibrated at all magnifications for size measurements. *Tg(ins:GFP)* embryos were imaged with an EVOS light cube GFP filter, using optimized light, exposure, and gain parameters for each age and magnification. The pancreas is located on the right lateral side of the zebrafish body; embryos and larvae were positioned optimally for visualization of the pancreas, and images have been mirror-flipped in Adobe Photoshop to account for inverted image acquisition. To analyze islet images, islet perimeters were manually traced after blinding the samples and the area was calculated in the EVOS software. For the tBOOH experiments, eleutheroembryos were imaged on an Olympus compound fluorescence microscope with a Zeiss monochrome camera Axiocam 503, with Zen acquisition and analysis software. Because this is an upright microscope, embryos were positioned to image on the right ventral side.

### 2.5. Statistics

Quartiles were provided for experiments assessing variability, and graphed using R software v.3.1.1. Interquartiles ranges (IQRs), defined as the range of values encompassing the first through the third quartiles, are presented along with the median values. A Mann–Whitney U Test was utilized for experiments investigating natural variability or toxicant exposures, using a confidence level of 95%. Variant data from the cyclopamine, MEHP, and PFOS experiments is presented as the number of affected embryos.

## 3. Results

### 3.1. Quantification of Islet Area among Treatment Control Embryos

**Figure 1:**
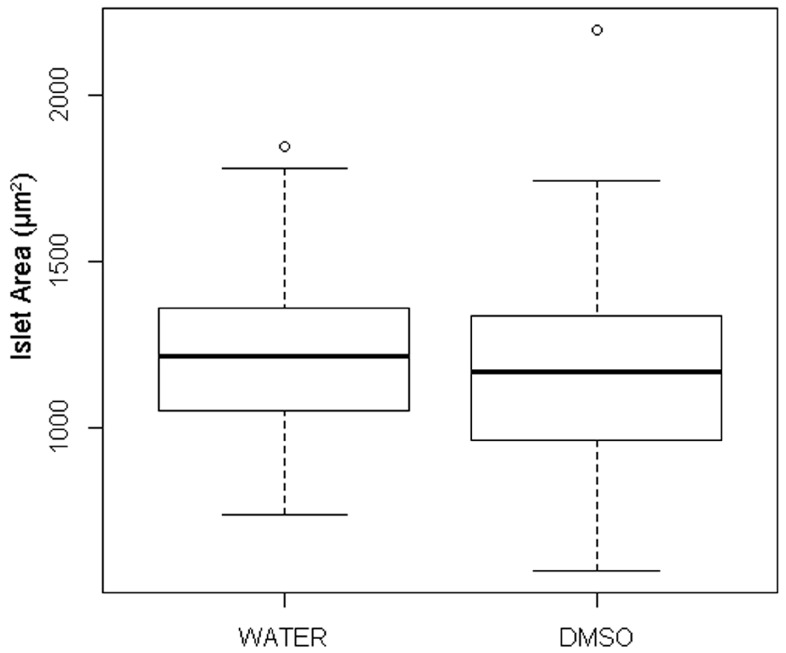
Embryos grown in dimethyl sulfoxide (DMSO) have similar islet sizes to embryos grown in water only. Islet areas were measured at 96 hpf in embryos raised in eggwater or in 0.01% DMSO, renewed daily. There was no significant difference in median area (*p* = 0.17) or distribution of islet areas between these control groups, though the range of islet areas for DMSO controls was slightly larger due to a high statistical outlier in the DMSO group (*n* = 44 embryos for the water group, and 72 for DMSO).

The most common vehicle used in zebrafish developmental toxicology experiments is dimethyl sulfoxamine (DMSO). To first characterize how a low, nontoxic concentration of DMSO may influence islet development, we exposed *Tg(ins:GFP)* embryos to a concentration of 0.01% *v*/*v*, refreshed daily, and compared islet size of DMSO-treated embryos to those maintained in egg water only, at 96 ± 2 hpf (Figure 1). Embryos maintained in egg water had a median islet area of 1216.39 µm^2^ (*n* = 44), while those maintained in 0.01% *v*/*v* DMSO had a median area of 1168.33 (*n* = 72). The first and third quartiles for embryos maintained in egg water only were 1055.98 and 1347.90 µm^2^ respectively, producing an IQR of 291.91. Those in DMSO had an IQR of 367.05, ranging from 961.93 to 1328.99 µm^2^. There was no statistically significant difference of islet size between untreated and DMSO treated embryos (*p* = 0.17).

**Figure 2:**
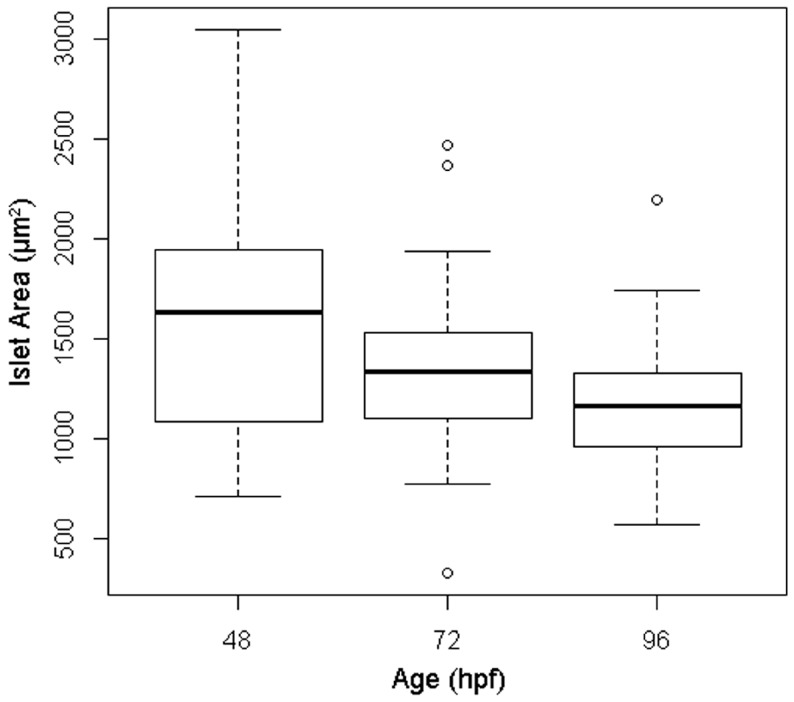
Islet size and variability changes over time during development in DMSO treated embryos. Islet size was measured at 48, 72, and 96 h post fertilization in embryos maintained in 0.01% DMSO, renewed daily Variability decreased between 48–96 hpf with the decreasing size of the islets, and assumed a more normal distribution. The highest range of area measures was observed at 48 hpf, while the lowest area variability was found at 96 hpf. The characteristic (normal) decrease in islet size from 48 to 72 hpf was not significant (*p* = 0.06), though the total decrease from 48 to 96 hpf was statistically significant (*p* < 0.001); *n* = 68, 42, or 72 embryos for 48, 72, and 96 hpf respectively.

Inter-individual variability is an important consideration for toxicological studies. Because our previous work identified variants in islet size among DMSO control embryos [14], we quantified the distribution of islet areas at 48, 72, and 96 hpf in embryos treated with DMSO (Figure 2). Islet areas characteristically decreased during this period due to structural condensation into a dense, spherical cluster of beta cells, with median areas of 1637.83 (*n* = 68), 1335.54 (*n* = 42; *p* = 0.06), and 1168.33 µm^2^ (*n* = 72; *p* < 0.001), at 48, 72, and 96 hpf, respectively. Variability of area also decreased over this period, with IQRs of 856.52, 418.67, and 367.05.

### 3.2. Identification of Islet Anomalies under Control Conditions

Several human pancreatic defects, such as pancreas divisum and ectopic pancreatic tissue, are predicted to occur in nearly 10% of the population [55,56,57]. We previously described examples of deviant development of the pancreatic islet, including fragmentation of the islet cell cluster, small or stunted islets, and the presence of ectopic beta cells (singular cells emerging outside of the primary islet, prior to secondary islet formation) [14]. While the majority of such anomalies were observed in PCB-126 treated embryos, some embryos in the DMSO control group also presented with aberrant islet morphologies [14]. For these reasons, a further investigation into the prevalence of islet structural variants and defects in islet development, particularly under the control conditions used in zebrafish embryotoxicity assays, was warranted.

**Figure 3:**
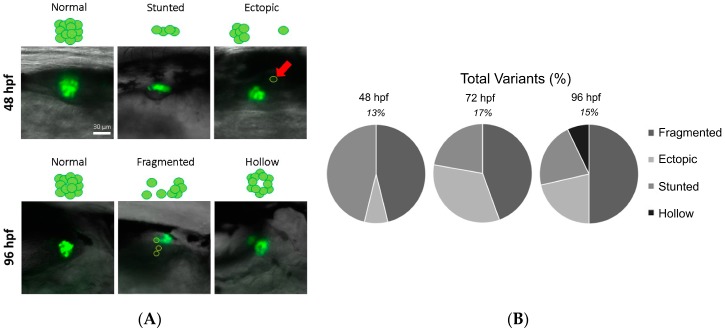
Islet variant morphologies observed within DMSO-treated embryos change throughout development. To complement the distributions of islet areas, islet morphologies were examined for morphological variants at 48, 72, 96, and 168 hpf. (**A**) Representative images of islet variants at 48 and 96 hpf are shown; (**B**) The prevalence of these variants changes over time; percentages shown are the total prevalence of variants among all DMSO-treated embryos. Pie charts show the distributions of each of the variant morphologies within the total number of variants at each time point. At 168 hpf, only one fragmented islet was observed; therefore, data variation could not be shown. All images are of the right lateral side, acquired with a 20 × objective, shown in a posterior (left) to anterior (right) orientation.

In the present study, embryos treated with a common concentration (0.01%) of DMSO were examined at 48, 72, 96, and 168 hpf for deviant islet morphology (Figure 3). At 48 hpf, 13% of embryos had variant islets (*n* = 9/67), namely fragmented islets (6%), stunted islets (6%), and ectopic beta cells (1%). At 72 hpf, 17% of embryos islets were variants (*n* = 9/53). Fragmented islets (8%), ectopic beta cells (6%), and stunted islets (4%) occurred. At 96 hpf, islet variants occurred in 15% of embryos (*n* = 13/89), namely fragmented (7%), stunted (3%), and hollow islets (1%), as well as ectopic beta cells (3%). To examine whether these morphologies persisted, primary islet variants were examined in the early larval stage at 168 hpf. The prevalence of variants was decreased to 2% (*n* = 1/52), at which time only one fragmented islet was observed.

### 3.3. Contaminant-Induced Abnormalities of the Primary Pancreatic Islet

Our study tested several contaminants that are classic teratogens and/or ubiquitous endocrine disrupting compounds during the critical windows of beta cell development, including cyclopamine, the phthalate metabolite MEHP, and the persistent perfluorinated compound PFOS. Here we provide examples of how chemical exposures can shift the occurrence of islet variants and defects, with an emphasis on the critical windows of exposure as well as the windows of observation.

#### 3.3.1. Cyclopamine

**Figure 4:**
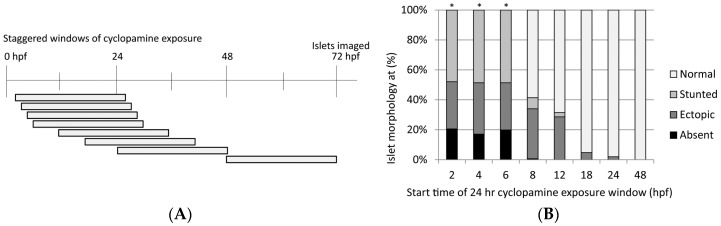
Beta cell defects caused by the steroidal alkaloid cyclopamine during embryonic development. (**A**) Design of cyclopamine experiment. Embryos were exposed to 50 µM cyclopamine in 28 °C embryo water for a period of 24 h beginning at differing stages of development (horizontal bars), then switched to embryo water alone and maintained until imaging at 72 hpf; (**B**) Islet morphology assessments in cyclopamine exposed embryos at 72 hpf. GFP + beta cells are normally organized into a compact cluster of cells located at the boundary of somite 4 during development. Embryos were assessed for presence of normal islets (presenting as compact spherical clusters), and morphological defects presenting in three categories: stunted islets comprised of a single cell, ectopic beta cells resembling an anterior islet duplication of single cells, and an absence of GFP + cells (no fluorescence). * These groups experienced 100% mortality by 72 hpf. Data are presented as the percentage of embryos exhibiting the islet categories within each exposure window. The number of embryos evaluated for each window of exposure were: 2–26 hpf 146; 4–28 hpf 152; 6–30 hpf 146; 8–32 hpf 150; 12–36 hpf 140; 18–42 hpf 105; 24–48 hpf 102; 48–72 hpf 100.

The timing of developmental exposures is important in determining the outcome of the endocrine pancreas morphology. We have previously demonstrated that exposure to the toxin cyclopamine disrupts sonic hedgehog (Shh) signaling and beta cell development in zebrafish embryos, resulting in islet morphologies that deviate from normal spherical clusters, and instead present as either stunted islets comprised of single beta cells, ectopic beta cells that resembled an anterior islet duplication, or absence of beta cells [15]. While exposure to cyclopamine also results in overt embryotoxicity ([15]), the effects on the islet are highly dependent upon the developmental stage at which the exposure occurred. In this experiment, developing embryos were exposed to cyclopamine, a potent inhibitor of Shh signaling, during different critical windows of embryogenesis. These windows were defined as a 24 h period starting and ending at staggered developmental stages starting as early as 2 hpf and as late as 48 hpf (Figure 4a). All embryos were then assessed at 72 hpf for effects on islet morphology. Normal morphologies were obtained when embryos were exposed to cyclopamine at or after 8 hpf (75% epiboly during gastrulation). The addition of cyclopamine during or before shield stage (6 hpf) completely abrogated normal beta cell development, as there were no normal beta cell clusters formed in the embryos treated before this time (Figure 4). These results demonstrate the importance of critical windows of islet development.

#### 3.3.2. MEHP

**Figure 5:**
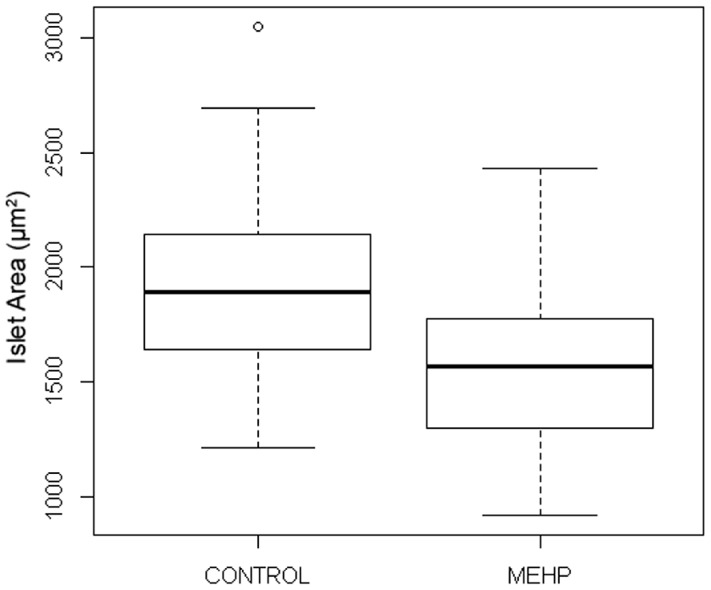
Developmental exposure to MEHP produces hypomorphic islets. Mono-2-ethylhexyl phthalate (MEHP) treatment decreases islet size in 48 hpf embryos. Median islet size was reduced by more than 10% in controls (*n* = 29) compared to MEHP-treated (*n* = 27) embryos (*p* = 0.001), and IQRs were similar and show a normal distribution of data.

In this study, we examined islet area and morphology in zebrafish embryos at 48 hpf following subchronic 0.7 µM MEHP exposure beginning at 3 hpf (Figure 5). There was no observed mortality or noted gross deformities. MEHP treatment resulted in significantly smaller islet areas than controls (*p* = 0.001). Control embryos (*n* = 29) had a median islet area of 1892.36 µm^2^, while MEHP islets (*n* = 27) had a median area of 1568.27. Control and MEHP-treated islets had similar IQR values, with 477.66 and 503.82, respectively. Incidence of islet variants and defects at 48 hpf was elevated compared to controls. Control embryos had a total incidence of 5/29, all of which were fragmented islets (Table 1). MEHP-treated embryos had a total variant and defect incidence of 7/27; 3/27 had ectopic beta cells, and 4/27 had fragmented islets.

#### 3.3.3. PFOS

**Figure 6:**
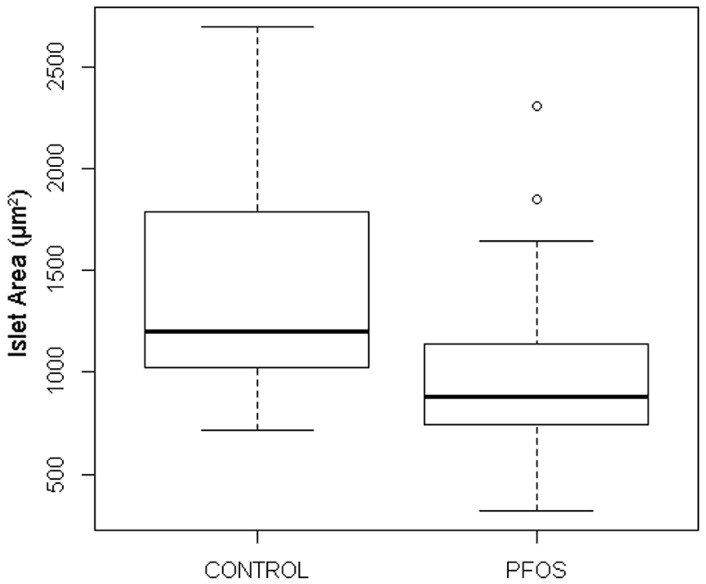
Embryonic Perfluorooctanesulfonic (PFOS) exposure decreases islet area. Subchronic PFOS treatment beginning at 3 hpf decreases islet area in 48 hpf embryos. PFOS-treated embryos (*n* = 39) were 26% smaller in area than control (*n* = 37) embryos (*p* = 0.001). Variability of control islet sizes Interquartile ranges (IQRs) was nearly double that of PFOS-treated embryos, though the IQR of these controls is similar to 48 hpf controls shown in Figure 2.

Islet area and islet morphological variants were examined at 48 hpf in zebrafish subchronically exposed to 32 µM PFOS beginning at 3 hpf (Figure 6). There was no observed mortality in either group, and no gross deformities were noted. PFOS reduced zebrafish beta cell mass in the primary islet at 48 hpf (*p* = 0.001). Control embryos (*n* = 30) had a median islet area of 1197.78 µm^2^, while PFOS-treated embryos (*n* = 33) had median areas of 882.61 µm^2^. The IQR of the control data was similar to the background IQR presented in Figure 2, with an IQR of 754.10. PFOS-treated embryos had a smaller IQR of 397.40. PFOS also increased the incidence of islet variants and defects at 48 hpf. Control embryos had a total incidence of 3/37, while PFOS-treated embryos had an incidence of 15/39. In controls, the incidence of fragmented (1/37) and stunted (1/37) islets, as well as ectopic beta cells (1/37) was low (Table 2). In PFOS-treated embryos, incidence of ectopic beta cells was still low (1/39), but the incidence of stunted (11/39) and fragmented (3/39) islets was elevated (Table 2).

#### 3.3.4. tBOOH

**Figure 7:**
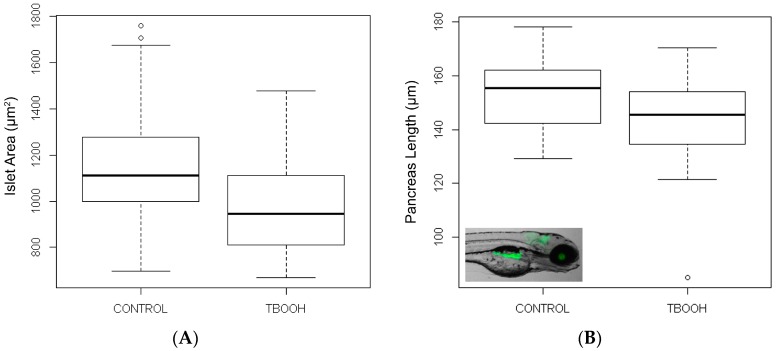
Treatment with the pro-oxidant *Tert* butylhydroperoxide (tBOOH) beginning at 72 hpf decreases islet area and pancreas length. Eleutheroembryos exposed to tBOOH manifested a significant decrease in islet areas and length of exocrine pancreas, measured at 96 and 80 hpf, respectively. (**A**) Median islet area for tBOOH treated embryos was 15% lower than that of water controls (*p* = 0.021); (**B**) tBOOH treatment at 72 hpf produced significantly shorter pancreata. Median pancreas lengths for tBOOH treated embryos was 6% shorter than controls (*p* = 0.038).

Islet areas were measured in embryos treated with tBOOH at 72 hpf (Figure 7A). There was no observed mortality in either the control or tBOOH-treated groups at the conclusion of experiments on 96 hpf. This is a period during which beta cells in the islet undergo substantial proliferation. tBOOH-treated embryos had decreased islet areas compared to control embryos (*p* = 0.021). The median control islet area (*n* = 27) was 1112.00 µm^2^, with an IQR of 266.15. tBOOH-treated embryos had a median islet area of 944.42 µm^2^, with a similar IQR of 266.04.

Pancreas length was also quantified in embryos following exposure to tBOOH beginning at 72 hpf. This is a period of rapid exocrine pancreas growth, as the pancreas nearly triples in length as it extends posteriorly. Treatment with tBOOH during this time significantly decreased pancreas length (*p* = 0.038). Median pancreas lengths for control embryos (*n* = 21) was 155.32 µm, with an IQR of 19.87. Median tBOOH-treated embryos (*n* = 13) had median pancreas lengths of 145.64 µm, with a similar IQR of 19.66.

## 4. Discussion

The developmental origins of health and disease paradigm postulates that later-life effects such as diabetes and metabolic disease could be due to effects caused by early-life conditions and chemical exposures [5]. Several environmental toxicants have been found to be associated with the development of diabetes in epidemiological studies (reviewed in [58,59,60,61,62,63,64,65,66,67]) or to disrupt the pancreatic beta cells in animal models [9,14,68,69,70,71,72,73]. However, in order to determine whether this is an associative or a causal relationship, additional laboratory studies are necessary. The data presented here demonstrate that the zebrafish embryo model is a valuable tool for detecting an important step in this developmental origins paradigm, and is capable of measuring subtle morphological variants and defects of the pancreas resulting from chemical exposures. We provide baseline incidence of control group morphology variants, and examples of developmental exposures to toxicants that result in disruption of pancreas morphology. This is a key step toward determining whether these toxicant exposures in the developing vertebrate embryo are linked to the developmental origins of metabolic dysfunction.

It is important to consider the genetic, phenotypic, and physiological diversity that naturally exists within a population. In this study, we observed that the distribution of islet areas among zebrafish embryos narrowed between 48–96 hpf. This is a very dynamic window of pancreas development, during which the two pancreatic anlages fuse, proliferate, and form a cohesive cluster of beta cells in the primary islet. During this window, the number of pancreas variants also tends to decrease with developmental stage. This decrease may occur due to the islet’s ability to rectify these aberrant morphologies as organogenesis progresses. Additionally, the absence of any stunted islets after 72 hpf without any loss of viability may be indicative of induced proliferation or catch-up growth. In epidemiological studies, “catch-up” growth is associated with an increased risk of cardiovascular and metabolic diseases later in life [74,75,76]. In future studies, we can further investigate potential islet “catch-up” growth and identify whether these islet variants have lasting phenotypic or metabolic effects.

We identified sensitive windows during which islets are especially susceptible to toxicant-induced damage, and assessed islet variant incidence rates. We have provided evidence that several toxicants produce deviant development of the primary islet, and that sensitivity to these exposures may change over time. Newly fertilized embryos treated with cyclopamine had increased incidence of islet defects, as we have previously shown [15]. However, those treated during late gastrulation and after had much lower incidence of aberrant islets. Early MEHP and PFOS treatment beginning at 3 hpf and maintained until 48 hpf also produced islet morphological variants, though these exposures were subchronic and did not test whether shorter windows throughout this timeframe produced different islet effects. In a previous study, we showed that administration of PCB-126 beginning later in development at 24 hpf decreased islet area and increased incidence of morphological variants [14]. While this exposure window follows key periods of early signaling pathways (such as hedgehog), it falls during a period of rapid beta cell proliferation and cell migration. Interestingly, these exposed embryos also showed increased incidence of islet anomalies, indicating that the window of beta cell susceptibility to toxicological perturbation may continue further into pancreatic organogenesis. This is further supported by the decreased islet areas observed due to tBOOH treatment here, despite being exposed to tBOOH only between 72–96 hpf. Therefore, it is possible that there are many windows of islet toxicological susceptibility, but each of these windows may be able to produce differing morphological effects.

**Figure 8:**
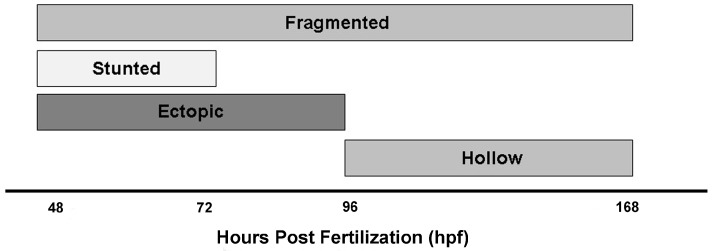
Islet variants occurrence differs with stages of development. Types of islet variants are only observed during specific windows of development. Stunted islets are only observed through 72 hpf, and ectopic cells until 96 hpf. Hollow islets are not observed until 96 hpf, but continue to be observed through 168 hpf. Across the experiments presented, fragmented islets appear to be the only variants observed across the entire developmental window investigated (48–168 hpf).

We identified specific windows of development during which particular islet variants appear (Figure 8). The most common variant observed, fragmented islets, occur throughout the entire window of embryonic development studied. However, stunted, ectopic, and hollow islets only exist during specific ranges. Stunted islets have only been observed in studies through 72 hpf, but none are visible at 96 hpf. Ectopic cells are only witnessed in our model through 96 hpf. Hollow islets are not observed prior to 96 hpf. The timing of these morphologies should be considered for toxicological studies, as some toxicants may only predispose embryos to specific variant morphologies. In this study, we did not observe any islet migration defects, such as those we identified previously with exposure to PCB-126. In that study, at 96 hpf, we observed the islet had migrated anteriorly, aligning with the third somite instead of the fourth, in addition to hypomorphic islets, fragmented islets, and ectopic beta cells [14].

In this study, exposures to cyclopamine, MEHP, and PFOS all increased the prevalence of at least one islet morphological variant. Though we do not yet fully understand the mechanisms which contribute to such anomalies, it is likely that the mechanisms to produce each of these anomalies are distinct. Here, MEHP increased the prevalence of ectopic beta cells in 48 hpf embryos, while PFOS increased the prevalence of stunted islets at the same age. Screening for similar and dissimilar variant patterns between classes of toxicants may help to identify the mechanisms behind each of these morphologies.

We identified 48 hpf as a sensitive islet assessment timepoint, during which a broader range of sizes and morphologies were observed for control conditions. Because of the dynamic morphological changes occurring in the pancreas at this time, this is likely to be a window of developmental sensitivity. The incidence and distribution of these variants at 48 hpf was exacerbated by toxicant exposures, indicating that beta cell development and islet morphogenesis is susceptible to perturbation before and perhaps during this period. Further study of more acute exposures during this early developmental period is necessary to improve our understanding of windows of differential islet susceptibility to toxicants throughout pancreas organogenesis.

In this study, we demonstrated that tBOOH exposures beginning at 72 hpf could shorten pancreas length 8 h later at 80 hpf. This is a period of dynamic exocrine pancreas growth, as the pancreas nearly triples in length. This exocrine pancreatic tissue will be responsible for the secretion of a number of digestive enzymes including proteases and lipases, and thus deviant development of the exocrine pancreas function may have lasting metabolic consequences. As with the endocrine pancreas, discrete windows of increased susceptibility to toxicological perturbation are likely to exist for the exocrine pancreas tissue, and thus acute exposure studies could better help to identify these critical timeframes.

Pancreas development is highly conserved between zebrafish and mammals. The developmental events guiding the early differentiation of pancreatic cells are initiated at similar phases of embryogenesis, and are directed by many of the same developmental signaling pathways [77,78]. Structurally, the zebrafish pancreas is similar to that of humans, though the separation of endocrine and exocrine tissue into independent pancreatic buds until 48 hpf (rather than mixed endocrine and exocrine cell populations in both buds) is specific to the zebrafish [79,80]. This spatial separation of endocrine and exocrine lineages may be utilized as a tool for toxicological research, allowing us to probe the localization of toxicity to either the exocrine or endocrine cell types, instead of a mixed cell population. This can then be used in toxicological screening studies to inform expected target tissues or cell types across vertebrate models.

Our understanding of the relationship between pancreas morphology and function is evolving. There is some evidence that supports that deviant islet morphology may have important significance for glucose homeostasis and metabolic function. We have shown previously that an increased prevalence of deviant islet morphologies is associated with altered expression of islet hormone genes in zebrafish embryos exposed to PCB-126 [14] and PFOS (under review). Also, there are several congenital pancreatic anomalies associated with increased risk of diabetes in humans that are similar to those we have observed in zebrafish embryos exposed to toxicants. These include pancreas divisum (failure of pancreatic buds to fuse resulting in two smaller pancreases [81]), dorsal pancreatic agenesis (short pancreas that lacks most islets) [82], ectopic pancreatic tissue [83], and annular pancreas (pancreas encircles the duodenum) [81]. These deformities have not been linked to genetic causes, and their etiologies are largely unknown, with the exception of annular pancreas which has an increased incidence in children whose mothers suffered from gestational diabetes [84]. Though some of these congenital abnormalities are very rare, others such as pancreas divisum and ectopic pancreatic tissue have been estimated to occur in as much as 10% of the population [55,56,57]. We have observed incidence rates of variant islets in zebrafish that are similar to background pancreas defect rates predicted for humans, and have measured increased incidence of these anomalies in zebrafish resulting from toxicant exposures. Given the conserved development of the pancreas between humans and zebrafish, it is logical that exposure to contaminants found to result in pancreas deformities in the zebrafish may result in similar deformities in human fetuses. Further, these conditions are rarely diagnosed in humans, as they may manifest as either asymptomatic or be masked by other phenotypes including pancreatitis and diabetes [57,85,86,87,88,89,90,91]. However, no studies have characterized whether these congenital defects are causal of these co-pathologies. The use of longitudinal transgenic zebrafish studies will allow us to directly observe whether embryos with variant phenotypes are more predisposed to these diseases later in the lifecourse.

In a diverse population, we would expect that natural diversity would give rise to variability of pancreas morphology and function in humans. In zebrafish, the variation of islet size and even structure indicate that the similarly developing human pancreas may also be highly variable. It is possible that these subtle developmental changes may produce lasting consequences, such as predisposition to metabolic dysfunction. The increased incidence of these variants in toxicant-exposed embryos suggests that there may be subpopulations with increased susceptibility. Though the embryos utilized in this study were from a variety of clutches, the contributions of genetics are unlikely to account for these differences. Further, the prevalence of these variants decreased over time, potentially due to corrective or catch-up developmental efforts. This suggests that there is the potential for the embryonic environment to not only influence the formation of these morphological anomalies, but also their remediation. More work is needed to determine why some of the eleutherolarvae are able to overcome these deformities in order to best identify preventative and potentially therapeutic measures that may be utilized for humans.

One of the remarkable features of the zebrafish model is its ability to regenerate, including regeneration of beta cells following targeted chemical ablation of these cells as adults (e.g., [92]). Because of this regenerative capacity, we are currently expanding this study to determine the persistence of islet deformities beyond the embryonic period, whether this regenerative capacity is compromised by early life toxicant exposures, and the functional consequences for glucose homeostasis. In addition, the formation and anatomy of secondary islets along the pancreatic tail that lines the intestine of the adult zebrafish may prove to be an important endpoint to characterize [92].

In conclusion, this work provides an experimental framework that contributes to the study of developmental origins of health and disease paradigm, with specific implications for metabolic diseases including diabetes. We have characterized the variability of the developing islet in the zebrafish embryo under typical control conditions used in embryotoxicity studies, and identified the ability of toxicants to produce potentially detrimental variant islet phenotypes within the first four days of development. This framework can be used to further investigate the mechanisms underlying toxicant impacts on the pancreas during development, and the relationship to phenotypic consequences or disease susceptibility later in life.

## Figures and Tables

**Table 1:** Embryonic MEHP exposure increases the incidence of islet variants at 48 hpf.

Variant Phenotype	Control (DMSO ^1^)	MEHP ^2^ (0.7 µM)
Fragmented Islets	5/29 (17%)	4/27 (15%)
Ectopic Beta Cells	0/29 (0%)	3/27 (11%)
Total Variants	5/29 (17%)	7/27 (26%)

^1^ DMSO, dimethyl sulfoxide; ^2^ MEHP, Mono-2-ethylhexyl phthalate.

**Table 2:** Developmental PFOS exposure increases the incidence of deviant islet morphologies observed at 48 hpf.

Variant Phenotype	Control (DMSO)	PFOS ^1^ (32 µM)
Fragmented Islets	1/37 (3%)	3/39 (18%)
Ectopic Beta Cells	1/37 (3%)	1/39 (3%)
Stunted Islets	1/37 (3%)	11/39 (28%)
Total Variants	3/37 (8%)	15/39 (38%)

^1^ PFOS. Perfluorooctanesulfonic acid.
